# Rupture, repair, the loss and re-construction of identity: Seeking support in situations of adolescent-to-parent violence and abuse

**DOI:** 10.3389/frhs.2023.1139727

**Published:** 2023-03-16

**Authors:** Nikki Rutter

**Affiliations:** Department of Sociology, Durham University, Durham, United Kingdom

**Keywords:** child-to-parent violence, violence and abuse, family violence, help-seeking, interpretive phenomenological analysis, motherhood

## Abstract

Family violence is associated with life-long implications including increased vulnerability to poor mental and physical health, and increased risk of repeat victimization. When children or adolescents are the ones instigating the harm, mothers experience the compounding issues of violence, mother-blame, and stigma. In comparison to other forms of family violence, how mothers understand and interpret their experiences of adolescent-to-parent violence and abuse (APVA) is underexplored, particularly in relation to what it means for them at the emotional and individual level, and how this impacts their sense of self, and their mothering and professional identities. This brief research report uses an interpretive phenomenological approach utilising hermeneutics to explore how six mothers made-meaning of their lives and identity when their parenting journey was disrupted by APVA. Help-seeking behaviours were often met with denial, avoidance, and parent-blame from professionals, unless they were familiar with the mother through her professional identity first. Neurodivergences of the adolescents were reported, including mental illness, autism, pathological demand avoidance, and foetal alcohol spectrum disorder. and as no mother reported successful engagement with social care, youth justice, or mental health services when help-seeking, they needed to reimagine their parenting role, or hit crisis, before accessing appropriate support. Mothers could have been supported earlier if critical incidents were identified by services, and support and/or interventions provided earlier, when mothers first engaged in help-seeking behaviours.

## 1. Introduction

Adolescent-to-parent violence and abuse (APVA) can cause both acute and chronic harm to families. Like other forms of family violence, it is associated with repeat victimisation, and increased likelihood of physical and mental illness throughout the lifespan (1). The aetiology of APVA is complex, although those young people who have experienced harm appear to be more vulnerable to instigating it themselves (1, 2). Due to the lack of existing knowledge around how APVA relates to family power dynamics and processes, it is inappropriate to apply existing models of domestic abuse to this phenomenon, and it requires distinct responses (2, 3). Thus, how parents living with APVA understand, interpret, and make meaning of their experiences could help identify appropriate pathways to support and intervention.

The purpose of this current work was to explore how parents who have experienced APVA make meaning of those experiences, as mothering through APVA is not only harmful due to the APVA itself, but also due to these experiences being compounded by layers of parent-blame and societal rejection (1). This blame and rejection is associated with the stigmatisation of APVA, whereby mothers experience dissonance between their experience of motherhood, their mothering identity, and the harmful behaviours or attitudes of their adolescents (4, 5). This mothering stigma is constructed both externally (judgement), and internally (self-stigma) whereby “parent self-stigma is best operationalised as including self-blame, self-shame, and bad-parent self-beliefs” (6).

It can also be challenging for mothers to seek support due to parental determinism, whereby parents are considered wholly responsible for the behaviour of their children, thus any behavioural challenges or difficulties must be the “fault” of the mother (6, 7). This is compounded by violence, as family violence rhetoric often blames the mother for not having more control when she is attempting to resist or navigate various realms of coercion (8). These parental blaming responses can result in mothers being blamed for APVA which perpetuates victim/mother-blaming narratives, and also holds mothers to account for the aggression of their child(ren), rather than questioning how services could improve their responses to disclosures of APVA. When mothers consider themselves a “good mother”, but experience mother-blaming discourses due to their experiences of APVA, this produces dissonance which can rupture the mother-child relationship (8–10).

## 2. Method

### 2.1. Participants

All participants (*n* = 6) were white British mothers who self-identified as a parent of a child (aged over 12) who had initiated violence towards a parent on a regular basis at some point during their parenting journey. There was also a sign language interpreter involved in the research (*n* = 1). All mothers were self-selecting after seeing a connected piece of research advertised on social media (11), or I contacted them directly due to pre-existing personal and professions connections. There were a mixture of current and retrospective narratives, and pen portraits are available for each participant which provides their pseudonym, family make-up, direction and time of APVA experience, and any disability of the parent or child identified.

#### 2.1.1. Pen portraits

##### 2.1.1.1. Amy

Amy is a single parent to a 13 year old daughter, who had initiated APVA for the previous 7 years. Amy is a birth parent, and identified that her daughter has a pathological demand avoidant (PDA) profile (considered a part of the autism spectrum which is underpinned by high anxiety and low tolerance for demands). Amy was recruited through seeing the research advertised by a PDA charity.

##### 2.1.1.2. Diane

Diane became a single parent to a son and daughter when they were aged 7 and 5 respectively, through adoption. They are now aged 18 and 16, and both have instigated APVA towards Diane and have continued to do so. Both children are autistic, one also has a learning disability. Diane saw the research advertised on a parenting support page for children with emotional and mental health needs.

##### 2.1.1.3. Evelyn

Evelyn is a mother to three children and is in a long-term relationship. Her middle daughter began initiating APVA at aged 12, which escalated until she was taken into care, aged 15, experiencing significant mental health difficulties. Evelyn was recruited through pre-existing affiliations and not a parenting support group for neurodivergences.

##### 2.1.1.4. Carol

Carol is married to David, and they are parents to two daughters, the eldest of whom is 20 and initiates APVA. At 10 months old, Carol's eldest daughter was diagnosed with cerebral palsy, and later on received a diagnosis of autism. Carol saw the research advertised on a parenting support page for children with emotional and mental health needs.

##### 2.1.1.5. Fiona

Fiona became a single parent to two boys when they were three and four years old through adoption. They are now 20 and 21 years old respectively and APVA started when they were aged 10/11. Both were taken into care aged 15 and 16. Fiona is now in a long-term relationship and is deaf, and used speech to communicate, however an experienced sign language interpreter, who Fiona had worked with previously, was provided to interpret interview questions. Fiona was recruited through pre-existing affiliations, not a parenting support group for neurodivergences.

##### 2.1.1.6. Rachel

Rachel is now retired and widowed. A birth parent to three children, Rachel experienced APVA when her son was aged 14–16 and this was directed towards his father, Rachel's husband. Rachel is a Christian, and regularly attends church. Rachel was recruited through pre-existing affiliations and had not accessed support for APVA or neurodivergences *via* support groups.

### 2.2. Approach

This brief research report is a follow-up piece from a larger piece of research into the early-stage dynamics of APVA (11), whereby it was clear that the experiences of violence were part of a much broader life story. I was interested in uncovering how mothers who have lived with APVA made meaning of their experiences in a way which attended to both the hermeneutics and phenomenology of their experiences. Thus, interpretive phenomenological analysis (IPA), which is an integrated, reflexive approach balancing description and interpretation to understand how individuals make meaning of their lived experiences, was an appropriate lens through which to explore the experiences of mothers (12).

### 2.3. Procedure

Eight semi-structured interviews, lasting between 60 and 150 min were conducted for this project. Participants were all provided with the first prompt “tell me about your parenting journey”, and then responses were participant-led with conversational prompts and inquiries from myself. Three interviews were held in person, two in the home of a participant, one in a community location; two interviews *via* phone, and three interviewers *via* Zoom. All interviews were recorded on an Olympus LS-P1, and all recordings were all transcribed by me. Two of the six participants were interviewed twice to clarify points made during their original interviews.

### 2.4. Ethical considerations

Durham University Department of Sociology granted ethical approval for the work. I have extensive practice and research experience in this field, which meant I could respond sensitively to the topic, and I shared a previous article written on a related topic with the participants prior to the work so they could see my approach to APVA, and decide whether this aligned with what they hoped for their own story. All participants gave written informed consent to participate in the work after reading an information sheet about the research. All were aware of their rights to withdraw.

### 2.5. Analysis

All analysis was conducted independently by myself as the sole author, and sense-checked by Fiona and Rachel. There were three levels of analysis, in line with an in-depth IPA approach (13). Firstly, I read, re-read, and listened to the audio files, made memos, notes, and engaged in reflexivity through the use of a research diary to critique my own interpretations. Secondly, I translated the notes made into thematic concepts engaging in the ‘hermeneutic circle (12, 13). This process promoted a reflexive approach to both how I interpreted the interpretations of participants, but also how I interrogated these interpretations in a dynamic way (13). Finally, I developed conceptual groupings consisting of themes and subthemes. It is these themes which are the focus of this paper.

## 3. Results

The themes identified were: identity (subthemes of professional and mothering identities); rupture; and repair. Each of themes demonstrated critical incidents experienced by mothers when they were help-seeking for themselves, or their child, when experiencing APVA.

### 3.1. Identity, professionalism and motherhood

There was tension between the mothering identity and the professional identity held by three of the mothers experiencing APVA and when professionals knew mothers as professionals first, and their personal, mothering identity second, this offered protective factors from mothering-blame discourses. For instance, Amy was an experienced health visitor:

*I was doing parenting courses just to tick that box to say, Yes, I've done that, I've been very open to support because I work closely with Children's Services anyway. I wasn't threatened by them. And a lot of the social workers, I kind of knew anyway, so I've got confidence in the way that they knew how I how I was as a Health Visitor, and how I helped families*.

However this protection was only relevant when mothers were known by support providers in their professional role, as holding a professional identity alone was not enough. If professionals were only viewed through the identity of “mother”, as was Fiona who was a social worker in an adults team, there were issues when help-seeking through safeguarding services. During one interview, Fiona's sign language interpreter provided information about her own observations as Fiona's sign language interpreter at meetings with social care and other services, with Fiona's consent:

*I've worked with Fiona for many years through home-life experiences *…* each agency that she interacted with blocked her from speaking about her experience. Social work, children and families. At any of the reviews, she was completely blocked… in a very hostile environment, she was not allowed to talk about her abuse… she was blocked every time and not allowed to talk about her abuse*.

When seeking support, Diane was a highly experienced healthcare practitioner, but she sought less support from professional services, and more from her employer. This was an effective strategy, as her employer viewed her through the identity of professional, and would provide flexibility in the workplace. When viewed through the identity of mother, responses were less helpful:

*The social worker was sort of saying to him, You carry on behaving like this, you will go into prison. Now he his mindset still in jail. And that's what was set in his brain*

Diane also found challenges relating to expectations of the mothering identity and role, as her children had particular expectations, leading to APVA when those expectations were not met:

*[My daughter] can be verbally very aggressive… [she] decided that I haven't kept her safe, that I've allowed [my son] to do this to her and carry on and on, so I'm not worthy to be her mother. So she used to call me Mama, now she doesn't. And she can be very verbally unkind, like fuck off, go! A lot more intense and much more persistent*.

Similarly, by viewing Amy through a mothering identity, Amy's ex-husband, and father of her daughter, engaged in mother-blaming narratives; whilst other professionals saw her as capable and competent, her ex-husband saw her as creating behavioural challenges:

*She was a dream child going there. Which, of course, [her Dad would say the cause of her behaviours] is always my parenting*.

For the other three mothers in this research, they did not speak about professional identities, but emphasised the challenges of the mothering identity. Their expectations of a “good mother” and their “good child” sat in contrast to their lived experience. For instance, both Carol and Rachel referred to being a “hard mother”, with strict boundaries and expectations of their child(ren) which created dissonance when it did not provide the desired behavioural result. Rachel stated she was “stricter” with her son than most parents, she ruminated “I used to think, what have I done wrong” when the family ruptured and her son needed to leave home due to the APVA.

### 3.2. Rupture

The cause of the rupture within the family was found to be connected to multiple, complex, neurodivergent needs across five of the narratives. As mentioned, Amy was confident in her own capacity to parent due to her professional identity, and so she sought answers for why APVA was occurring, however, she felt she had failed both roles by not recognising that her daughter was autistic:

*[The autism] diagnosis came to visit real shock to me… I thought I'd let her down, because I'm a nurse, I'm meant to pick up on these signs. But the guy that… gave her the diagnosis. He said, You've just, you've adapted… you've adapted your language …  your approach to her from day one. Because you know what works … So that kind of reassured me really… Once I learned more about the process, and then I learned about PDA. My mind just exploded. It got a lot worse before it got better*.

PDA is understood as being avoidance, underpinned by anxiety (14). The unmet needs experienced by Amy's daughter, due to her neurodivergence, meant that Amy had to re-assess her parenting strategies. On the other hand, Carol recognised that her eldest daughter had needs beyond what could be understood through her cerebral palsy, but when seeking understanding of the APVA, Carol explained “most of her difficult behaviours have always been blamed on the cerebral palsy” resulting in Carol and her family being left without support, and increasing levels of APVA. Finally they received an explanation that the eldest daughter had needs that were not being met, but no direction regarding how to navigate this:

*I tried to get [support]when she was about 14, with this possessiveness … they just offered their CBT [cognitive behavioural therapy] … that didn't work at all. So we got to 17 and a half, and we had a particularly bad Friday afternoon… [eventually] they assessed her, sort of like, three weeks before her 18th birthday. Said, you know, yes, she's got autism, thank you very much goodbye*.

Similarly, whilst believing both of her sons had foetal alcohol spectrum disorder (one had a diagnosis), Fiona sought support from services but, despite experiencing significant physical harm, Fiona was unable to find an appropriate pathway to support herself or her sons:

*I was on my own, that the abuse started and I experienced it for nearly six years from [the eldest being 11] right to there. So, and I continually took them to the GP, the pediatrician, to other assessment clinics, CAMHS [child and adolescent mental health services], to try and find out if there was anything that the boys needed support with*.

All mothers evidenced that they sought support for their families in an attempt to identify what their needs were, as well as appropriate mechanisms for keeping everyone in the home safe and happy, but in four cases, the APVA escalated rather than reduced due to the lack of appropriate intervention, as demonstrated by Fiona:

*Holes in the walls got really all scattered up the hallway, up the stairway. And that continued and continued and the younger one actually just copied the behaviour. And glass got smashed. And I'm talking about from cabinet glass, cabinets from the kitchen units. So it's just continuous, absolutely continuous. And I think the one that really shocked me, was when the 11 year old was able to pull me along, he pulled me along the floor by my hair*.

When systems failed to respond to disclosures of APVA appropriately, there was evidence that the harm began to extend into other systems. Four mothers reported that school became part of the system, either due to authoritarian measures (Evelyn; Fiona), providing a pathway for their child to be exploited (Rachel), or not providing a suitable learning environment (Carol). This led to disruption and distress for the young person. For instance, if a child could not or would not attend school, mothers were receiving repeated contacts from school asking for the young person to be collected, supported, or reporting they had absconded. This made work and home-life difficult, as mothers were requesting support, but being asked to “control their child” in return, with three mothers leaving their jobs due to the needs of their child, as evidenced by Evelyn:

*I'm still asking for help and support. I then had to leave my job because she was walking out of school or attacking teachers. I just couldn't work. There's just no way*.

Four mothers reported that help-seeking resulted in escalating levels of APVA, rather than a reduction. When seeking support when in crisis Evelyn found that, when her daughter was experiencing a significant mental health crisis, threatening to kill members of the family, instigating significant levels of harm and using her bedroom as a toilet, the family were not supported by services. Instead, after assessment, Evelyn's daughter was returned home without a care package:

*She attacked her brother like, considerably. She was very sick, she would have been at this point, 13 or 14. She was removed from the property and I asked for her to stay away from the home due to what has happened. Before I was threatened with prosecution if I didn't take my daughter back; or removal of my other children. While I was forced to have her back, and then things took a very drastic turn. At that point. She was returned to our care and no safety plan, no risk assessment for my children, no support whatsoever*.

This left Evelyn unable to respond to the risks of harm her daughter posed within the family. Only one mother reported professional responses were helpful, Rachel, who found the involvement of the police, once her son had left home due to the escalating APVA, helped her understand that it was not her “fault” that APVA had occurred, or that her son had become involved in theft and substance misuse:

*So we explained to the police all of it that had happened. [my son] came twice a week for a meal and his washing and we gave him money every week … he was still supposed to be at school and the policeman said ‘Do you even know how lucky you are?”, he said we had a nice home we're a respectable, nice family*

### 3.3. Repair

Repair for the mothers was not about repairing the damage of poor relationships with services, but the ways they had attempted to repair the relationship with their child(ren). For instance, Amy, Carol, and Diane all found that changing their parenting approach to utilise more “autism strategies” reduced the frequency and intensity of incidents of APVA, as they were better equipped to meet the needs of their child(ren). As explained by Diane:

*After the diagnosis, and the fluoxetine and learning about that it was not her it was that emotions flying off … something was distressing to her. And so like being able to then modify everything. And then [my younger daughter] started to realize that you had to handle things in a different way. [David] took a long time to accept that life had to be different. And once all three of us were on board that she wasn't just a spoiled child, and that we were letting her get away with murder … Things got a lot better*.

This reframing meant that mothers expressed that the wider family were able to understand the explosive impulses or aggression through a diagnosis, and changed their own approach to be more understanding of their everyday difficulties of neurodivergence. However, Diane touched upon one of the compounding factors of having adopted older children, and the trauma that came alongside the autistic needs, the trauma, and the development aspects of adolescence:

*How much of it is development, trauma, and how much of it is autism? Because you're never on it completely. Because I think the older he gets, the more I can see and believe because I tend to take notic*e of the journey.

Two mothers reported their child(ren) had been accommodated through a voluntary care order (Section 20 care order). Although accessing this was a battle for both Evelyn and Fiona, with the Local Authority either stating that the child was the responsibility of their mother, or too high-risk to be taken into care, Fiona explained this was something that frustrated her, as when both boys were accommodated, they were considered too high risk to be placed together: “all that time I was asking for help and they said I should be able to manage by myself, but when they take them the risk was too high.” For both Fiona and Evelyn, once professionals were able to witness some of the harmful behaviours themselves, they began to believe the reports of APVA, as Fiona reported “both boys were absconding, coming back drunk and smoking drugs in their rooms.”

The time and space the Section 20 care order provided them allowed for some reconciliation, whereby they were able to have more positive relationships with their children. For Fiona it was accessing counselling, and learning about her identity as an individual, not as a mother or professional due to her needing to take “early retirement for [her] health”. Similarly, Evelyn made meaning of an intensely traumatic APVA experience which resulted in the forced removal of her daughter from the home by police, by using it as an opportunity to re-establish a healthier family:

*I said when she was removed from the house that this is the starting point, not the end. And I fully believed that. It was what was right for us in order for us to remain as a family and be safe to do. She needed not to be* home.

Only one mother did not get to experience a reconciled relationship with her child, Rachel. Despite her narrative being an account of events approximately 40 years ago, she continued to hope her relationship with her son would be repaired, initially through long-distance connection, right up until his unexpected death:

*[The year he died] he sent me a Mother's Day card. The first one I'd had for years, and he said to [his sister] that he was going to come home to see us and make amends. But when he had the pub, I went to the pub about three or four times so we were all right then … The Mother's Day card is in [the house] because I never parted with it. I just never parted with it*.

## 4. Discussion

Experiences of APVA being recognised and validated as harmful and not the “fault” of the mother was connected to whether the professional or mothering identity was seen first. Knowing mothers as professionals first, and their personal, mothering identity second, meant that mothers in professional settings were more likely to be protected from the mother-blame that has been identified in previous work (9, 10). Furthermore, this professional-protection from mother-blaming, may have assisted in reducing the ambiguity of APVA, as the description by professional mothers was validated (9). In contrast, when viewed as a mother first, there were more mother-blaming narratives, and barriers to accessing services or support.

Whilst some mothers attempted more authoritarian parenting practices, this style of parenting has been associated with higher levels of APVA (15), and many mothers had to change their strategies to reduce the frequency or intensity of the APVA (3). For four mothers, the behaviours began in pre-adolescence, challenging the idea that only adolescents engage in APVA to express their autonomies and freedoms (3). All mothers recognised the harm escalated in adolescence, when the young person engaged more with school and the community, which supports findings in similar work (16).

Previous IPA work on APVA found that the concepts of powerlessness within the parenting role made APVA somewhat ambiguous, and difficult to define (9). Furthermore, the same work identified that parents needed access to support pathways when experiencing APVA (9). However I did not find ambiguity or mothers unclear of how to ask for support. All mothers were help-seeking during critical incidents from mental health, social care, public health, and policing services without being referred for further risk assessments or intervention, and all of the young people fit at least one of the criteria that increases vulnerability to instigating APVA (2, 17). Thus, there are clear recommendations for services to improve their response to APVA in particular.

### 4.1. Limitations

As a short report, this work has not examined some of the nuanced or granular experiences of living with disability and neurodivergence which were only discussed in relation to APVA and education. Thus missing some of the overlapping and distinct complexities experienced by families living with neurodivergence and/or disability. Similarly, as this paper focused upon how mothers made meaning of their experiences of APVA, siblings, fathers, and wider family perspectives are not considered; holding the mother not responsible for the occurrences of APVA, whilst also not having opportunity to explore contextual factors which could increase risk and vulnerability.

### 4.2. Recommendations

Mother-blaming compounded the harms caused to mothers by APVA, and when mothers were viewed as professionals they experienced different responses from practitioners. Practitioners should be aware of this bias when working with families, and recognise their reports as potential recordings of escalating critical incidents. Whilst the mothers in this research sought support from mental health services, safeguarding teams, policing, and education, there was little to no evidence of how services worked together. Future research could further explore how the structural challenges experienced by help-seeking mothers living with APVA outlined in this article could have been better navigated if there were consistent multi-agency pathways to support.

## Data Availability

The raw data supporting the conclusions of this article will be made available by the authors, without undue reservation.
